# Optimizing Annealing Temperature for Enhanced Electrical Performance and Stability of Solution-Processed In_2_O_3_ Thin-Film Transistors

**DOI:** 10.3390/mi16101091

**Published:** 2025-09-26

**Authors:** Taehui Kim, Seullee Lee, Ye-Won Lee, Dongwook Kim, Youngjun Yun, Jin-Hyuk Bae, Hyeonju Lee, Jaehoon Park

**Affiliations:** 1 School of Semiconductor Display Technology, Hallym University, Chuncheon 24252, Republic of Korea; kth7953@hallym.ac.kr (T.K.); d.kim@hallym.ac.kr (D.K.); youngjun.yun@hallym.ac.kr (Y.Y.); 2 School of Nano Convergence Technology, Hallym University, Chuncheon 24252, Republic of Korea; sul0129@hallym.ac.kr (S.L.); yw330@hallym.ac.kr (Y.-W.L.); 3 School of Electronic and Electrical Engineering, Kyungpook National University, Daegu 41566, Republic of Korea; jhbae@ee.knu.ac.kr; 4 School of Electronics Engineering, Kyungpook National University, Daegu 41566, Republic of Korea

**Keywords:** indium oxide, thin-film transistor, solution-processed, Raman-spectroscopy, thermal annealing, hysteresis

## Abstract

This study investigates the influence of post-deposition thermal annealing temperature on the crystal structure, chemical composition, and electrical performance of solution-processed indium oxide (In_2_O_3_) thin films. Based on thermogravimetric analysis (TGA) of the precursor solution, annealing temperatures of 350, 450, and 550 °C were adopted. The resulting In_2_O_3_ films were characterized using ultraviolet–visible (UV–Vis) spectroscopy, atomic force microscopy (AFM), Raman spectroscopy, and Hall-effect measurements to evaluate their optical, morphological, crystalline polymorphism, and electrical properties. The results revealed that the film annealed at 450 °C exhibited a field-effect mobility of 4.28 cm^2^/V·s and an on/off current ratio of 2.15 × 10^7^. The measured hysteresis voltages were 3.11, 1.80, and 0.92 V for annealing temperatures of 350, 450, and 550 °C, respectively. Altogether, these findings indicate that an annealing temperature of 450 °C provides an optimal balance between the electrical performance and device stability for In_2_O_3_-based thin-film transistors (TFTs), making this condition favourable for high-performance oxide electronics.

## 1. Introduction

Transparent conductive oxides (TCOs) have garnered considerable attention as essential materials for next-generation electronic and optoelectronic devices owing to their high optical transparency and excellent electrical conductivity [1]. Among various TCOs, indium oxide (In_2_O_3_) has been thoroughly investigated because of its wide bandgap and intrinsically high carrier concentration that lead to superior electrical and optical characteristics. In_2_O_3_ adopts a bixbyite cubic crystal structure with a lattice constant of a = 10.117 Å [2]. This crystal structure provides a favourable pathway for high electron mobility due to which In_2_O_3_ is widely used as a transparent electrode material for various applications, including electrochromic windows, flat panel displays, organic light-emitting diodes, and solar cells [3]. In_2_O_3_ is known as a wide-bandgap semiconductor with a direct bandgap of approximately 3.7 eV and an indirect bandgap of around 2.6 eV. Although it typically appears as a yellow powder in the bulk form, it can be fabricated into thin films that maintain high transparency in the visible light range.

Such films can be deposited using various techniques, including reactive thermal evaporation, ion beam sputtering, direct-current reactive magnetron sputtering, pulsed laser deposition, and chemical vapour deposition. These techniques critically influence the crystallinity, electrical conductivity, and optical response of In_2_O_3_ films. Parameters such as the substrate temperature and oxygen partial pressure play pivotal roles in modulating the carrier concentration and mobility [4]. In addition to the deposition methods, post-deposition thermal treatments serve as a critical step in enhancing the structural integrity and electronic performance of oxide thin films. Post-treatments such as rapid thermal annealing and laser annealing can enhance device reliability by promoting grain growth, healing defects, and relieving internal stress. Annealing parameters such as the temperature and duration influence various factors such as dopant diffusion, oxygen vacancy control, and interfacial reactions with the substrate that ultimately affect the charge carrier dynamics and band structure within the film [5].

Solution-based fabrication of In_2_O_3_ thin films by sol–gel methods is emerging as a promising strategy for the development of next-generation flexible electronic devices owing to its low cost and compatibility with low-temperature processing. However, unlike vacuum-deposited films, solution-processed counterparts undergo fundamentally different growth mechanisms. This distinction highlights the necessity for comprehensive studies on the microstructural evolution and defect states under varying annealing conditions. For instance, vacuum-based deposition typically involves a top-down growth mechanism by nucleation on the substrate surface, whereas solution processing induces a bottom-up solidification process in which the solute is uniformly distributed throughout the film and undergoes densification from the bottom to top [6]. Meanwhile, ensuring the operational stability of thin-film transistors (TFTs) has become a critical challenge in display and sensor-based electronics, with particular attention paid to the hysteresis phenomenon that undermines the repeatability and reliability of device characteristics. Hysteresis refers to an irreversible electrical behaviour wherein the drain–current response varies depending on the gate voltage sweep direction.

This effect is mainly attributed to trap states at the semiconductor–dielectric interface, residual defects in the oxide film, or charge accumulation and leads to threshold voltage instability and degradation of current-level reproducibility, thereby compromising the long-term reliability of the device [7,8]. In high-resolution displays and flexible or wearable electronics, precise control of the operating conditions is essential. Hence, hysteresis mitigation is considered a vital aspect of device technology. Therefore, it is essential to identify the origins of charge trapping and interfacial defects in thin films and derive the optimal annealing conditions to suppress them. This can enhance the performance and long-term operational stability of In_2_O_3_-based TFTs [7,9]. In this study, In_2_O_3_ thin films fabricated by solution processing were systematically analyzed to investigate the influence of thermal annealing conditions on defect formation and hysteresis behaviour in relation to electrical properties. The objective of this study is to identify the optimal annealing conditions that enhance the thermal stability of In_2_O_3_ thin films, while suppressing charge-trapping phenomena. These findings provide a comprehensive foundation for the reliable operation of solution-processed In_2_O_3_-based TFTs.

## 2. Materials and Methods

**Figure 1:**
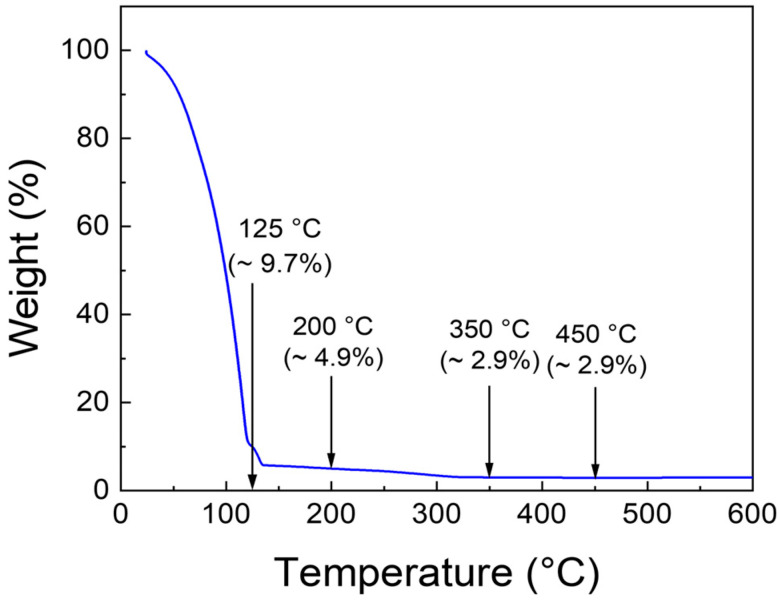
TGA characteristic curve of the prepared In_2_O_3_ precursor solution.

A 0.2 M precursor solution was prepared by dissolving indium nitrate hydrate (In(NO_3_)_3_·xH_2_O, 99.999%, Sigma-Aldrich, St. Louis, MO, USA) in 5 mL of 2-methoxyethanol anhydrous (CH_3_OCH_2_CHOH, 99.8%, Sigma-Aldrich, St. Louis, MO, USA). The solution was magnetically stirred at 350 rpm on a hot plate maintained at 75 °C for 5 h to ensure complete dissolution and compositional homogeneity. Thermogravimetric analysis (TGA) N-1000 system (Scinco, Seoul, Republic of Korea) was performed to investigate the thermal decomposition behaviour of the precursor solution. The measurement was carried out under a nitrogen atmosphere with a heating rate of 10 °C/min from 25 to 600 °C. The TGA curve of the precursor solution is shown in Figure 1.

A significant initial weight loss exceeding 90% was observed at approximately 125 °C, primarily due to solvent evaporation and precursor decomposition. Between 125 °C and 350 °C, the rate of weight loss decreased and eventually plateaued. As the temperature continued to increase, weight loss gradually slowed and stabilized as the temperature rose from 125 to 350 °C. In this temperature range, hydrolysis of In^3+^ species leads to the formation of indium hydroxide (In(OH)_3_). Above 350 °C, negligible weight loss was observed, which is attributed to the dehydroxylation of In(OH)_3_ and the subsequent formation of crystalline In_2_O_3_. The minimal mass change above 350 °C further confirms the thermal stability of In_2_O_3_ in this temperature regime. Notably, the thermal decomposition of In(OH)_3_ into In_2_O_3_ and H_2_O typically occurs within the temperature range of 340–600 °C [10,11]. Therefore, annealing temperatures exceeding 350 °C are sufficient to achieve complete conversion of the precursor into In_2_O_3_. Accordingly, annealing was conducted at 350, 450, and 550 °C to convert the precursor solution into a crystalline In_2_O_3_ thin film.

Quartz substrates were used for optical absorbance measurements. For AFM, Raman, Hall effect, and TFT devices, a 100 nm thick silicon nitride (SiN_X_) layer was deposited on p-type silicon wafers to function as the gate insulator. All substrates were first cleaned by sequential ultrasonication in acetone, isopropyl alcohol, and deionised water, followed by nitrogen drying and baking at 180 °C for 1 h. Oxygen plasma treatment (CUTE, Femto Science, Hwaseong-si, Republic of Korea) was subsequently performed for 1 min at a radio frequency power of 40 W with an oxygen flow rate of 20 sccm to enhance the surface hydrophilicity and improve the interfacial compatibility with the In_2_O_3_ layer. The precursor solution was filtered through a 0.2 μm polytetrafluorethylene syringe filter prior to deposition. Spin coating was performed on the oxygen plasma-treated substrates using an ACE-200 system (Dong Ah Trade Corporation, Seoul, Republic of Korea) at 5000 rpm for 35 s. All spin-coating experiments were performed under controlled laboratory conditions, with the relative humidity maintained at 40–45%. Measurements were not conducted when the humidity deviated from this range, minimizing variations in the resulting In_2_O_3_ thin films. For optical absorbance characterization, six samples were prepared: Sample A (bare quartz), Sample B (spin coating only), Sample C (spin coating followed by prebaking on a hot plate at 80 °C for 5 min), and Samples D, E, and F (spin coating and prebaking followed by thermal annealing in a box furnace (C-10P, Hantech Co., Gunpo, Republic of Korea) at 350, 450, and 550 °C, respectively, for 30 min in ambient air to induce the formation of In_2_O_3_.

The optical absorbance spectra of the thin films were obtained using a UV–Vis spectrophotometer (X-ma 3000PC, Human Corporation, Seoul, Republic of Korea) to evaluate the transparency and bandgap of the films. AFM measurements were performed on SiN_X_ substrates using the same deposition procedures as described for the optical samples. A total of five devices were fabricated: one device underwent only spin coating, another device underwent spin coating followed by prebaking, and the remaining three devices underwent spin coating and prebaking followed by thermal annealing at 350, 450, and 550 °C, respectively. AFM was carried out using (XE-N80, Park Systems, Suwon, Republic of Korea) to investigate surface morphology, film roughness, and thickness variation. Raman measurements were conducted on four devices following the same deposition procedures: one device was prebaked only, and the remaining three devices were prebaked and thermally annealed at 350, 450, and 550 °C, respectively.

Raman spectra were obtained using a LabRAM HR Evolution system (Horiba Jobin Yvon, Kyoto, Japan) to analyze the crystallinity and structural evolution of the In_2_O_3_ films. For Hall effect and TFT characterization, only the three thermally annealed devices (350, 450, and 550 °C) were used. Hall effect measurements were performed using a Hall measurement system (HMS-3000, Ecopia, Anyang, Republic of Korea) to determine bulk concentration and mobility. For the TFT fabrication, a bottom-gate/top-contact structure was adopted. An aluminum (Al) source and drain electrodes with a thickness of 140 nm were thermally evaporated using a shadow mask. The channel width (W) and length (L) of the TFTs were 2000 and 80 μm, respectively. The electrical performance of the In_2_O_3_-based TFTs was evaluated under ambient conditions with a probe station (Model 4000, MS Tech, Seoul, Republic of Korea) to determine output and transfer characteristics.

## 3. Results and Discussion

**Figure 2:**
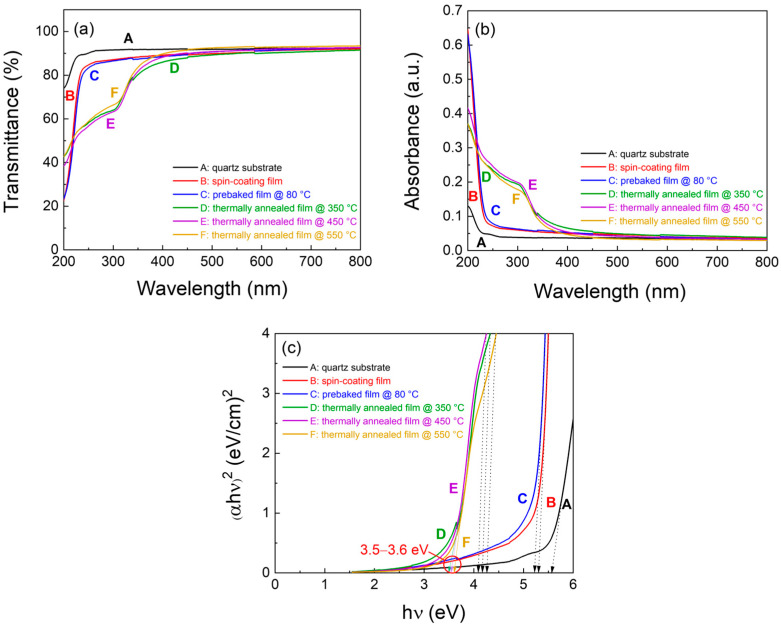
Optical (**a**) transmittance and (**b**) absorbance spectra of the solution-processed films. (**c**) Determination of optical band gap of the solution-processed films using Tauc’s plots.

To examine the influence of annealing temperature on the optical properties of the solution-processed In_2_O_3_ films, samples were deposited on quartz substrates under varying thermal treatments. The first film was deposited on a bare quartz substrate without any thermal treatment. The second film was prebaked at 80 °C, and the remaining three films were subjected to the same prebaking step followed by thermal annealing at 350, 450, and 550 °C. Ultraviolet–visible (UV–Vis) spectroscopy was employed to measure the optical transmittance of the films in the wavelength range of 200–800 nm. Figure 2a shows the transmittance spectra of the films as a function of wavelength. As shown in Figure 2a, the films exhibited a transmittance greater than 80% in the visible range from 400 to 800 nm, with the transmittance decreasing from 400 to 200 nm owing to absorption effects. The films that were only prebaked at 80 °C exhibited similar transmittance profiles, whereas those subjected to additional thermal annealing at 350, 450, and 550 °C showed nearly identical optical behaviours, suggesting that crystallinity or densification reached saturation above 350 °C.

Figure 2b shows the absorption spectra of the films as a function of wavelength. The bare quartz substrate, spin-coated film, and the film prebaked at 80 °C exhibited an absorption edge below approximately 350 nm. In contrast, the films annealed at 350, 450, and 550 °C exhibited absorption edges in the ranges of 300–320 nm and 260–320 nm, indicating enhanced absorption in the UV region. The optical band gap energy (*E_g_*) was estimated using Tauc’s relation, (αhν)^2^ ∝ (hν − *E_g_*), where α is the absorption coefficient, h is Planck’s constant, and ν is the photon frequency. The absorption coefficient (α) was calculated from the transmittance (T) using the relation: α = [2.303 × log(1/T)]/d, where d is the film thickness. Figure 2c presents the Tauc plots, i.e., (αhν)^2^ versus hν, from which *E_g_* was determined by extrapolating the linear portion to (αhν)^2^ = 0 [12,13]. The estimated *E_g_* values were approximately 5.5 eV for the bare quartz substrate, 5.3 eV for the spin-coated film, and 5.2 eV for the film prebaked at 80 °C.

**Figure 3:**
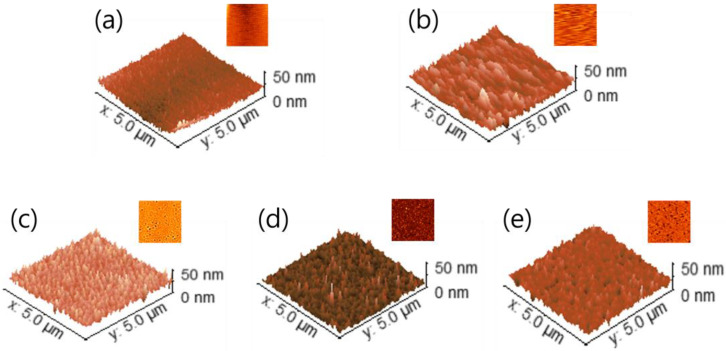
AFM images (5 μm × 5 μm) of the solution-process films fabricated through (**a**) spin-coating, (**b**) prebaking at 80 °C, thermal annealing at temperatures of (**c**) 350, (**d**) 450, (**e**) 550 °C.

These results suggest that the precursor films, including those processed by spin-coating and prebaking at 80 °C, did not convert to In_2_O_3_. In contrast, the films annealed at 350, 450, and 550 °C exhibited *E_g_* values of approximately 3.50, 3.54, and 3.60 eV, respectively, which were consistent with the direct band gap energy of bulk In_2_O_3_ (3.5–3.8 eV) [12,14]. Moreover, the absorption bands observed in the 260–320 nm range corresponded to the estimated *E_g_* values of approximately 4.00–4.30 eV, exceeding that of bulk In_2_O_3._ Previous studies have reported that the elevated E_g_ values can be attributed to the quantum confinement effect originating from the nanostructured characteristics of In_2_O_3_ films [6,15]. The surface morphologies of the solution-processed thin films were characterized using atomic force microscopy (AFM), which provided topographical images in two and three-dimensional modes. Changes in surface roughness, quantified by root mean square (RMS) values, were evaluated as a function of the fabrication and thermal annealing conditions. In general, during solution-based film formation, the solute species are distributed throughout the bulk and near the surface of the coated film. Substrate heating facilitates bottom-up solidification of the film [16]. This complex mechanism plays a critical role in determining the morphological characteristics of solution-processed thin films. Figure 3a–e show the grain structures and surface features of the prebaked films, suggesting that the grain growth and condensation behaviour are influenced by subsequent thermal annealing. Increasing the annealing temperature promotes crystallinity and induces condensation-related stress, which collectively contribute to the development of internal stress, thereby altering the surface morphology. The as-spin-coated film exhibits the lowest RMS roughness (1.99 nm), indicating the formation of a relatively smooth surface. After the prebaking process, the RMS value increases slightly to 2.09 nm, indicating the introduction of minor morphological variations during the solvent evaporation phase. In contrast, the thermally annealed films exhibit a progressive increase in surface roughness with increasing annealing temperature. The RMS roughness values are determined to be 3.82 nm at 350 °C, 4.03 nm at 450 °C, and 4.32 nm at 550 °C. This progressive increase in surface roughness can be attributed to surface coarsening resulting from the removal of residual organic components and growth of crystalline grains during thermal annealing.

While no significant increase in RMS roughness is observed above 450 °C, the AFM images reveal well-defined grain boundaries. These observations imply that the nanoscale grains initially formed during the prebaking stage underwent further agglomeration at elevated temperatures, leading to the formation of a more consolidated film structure. Overall, the AFM analysis demonstrates a clear trend of increasing surface roughness with increasing annealing temperature, suggesting that high-temperature thermal treatment enhances crystallization and induces stress-driven morphological evolution [17,18]. Additionally, the cross-sectional thicknesses of the In_2_O_3_ channel layers were measured using AFM under different thermal annealing conditions. The results indicate that the device annealed at 350 °C exhibited a channel thickness of 30 ± 1 nm, whereas devices annealed at 450 °C and 550 °C both showed a reduced thickness of 20 ± 1 nm. These results suggest that increasing the annealing temperature leads to densification of the In_2_O_3_ film and a corresponding reduction in channel thickness. The observed reduction in channel thickness at higher annealing temperatures is consistent with the increased surface roughness, indicating that thermal annealing promotes film densification while simultaneously enhancing crystallinity and inducing stress-related morphological changes.

**Figure 4:**
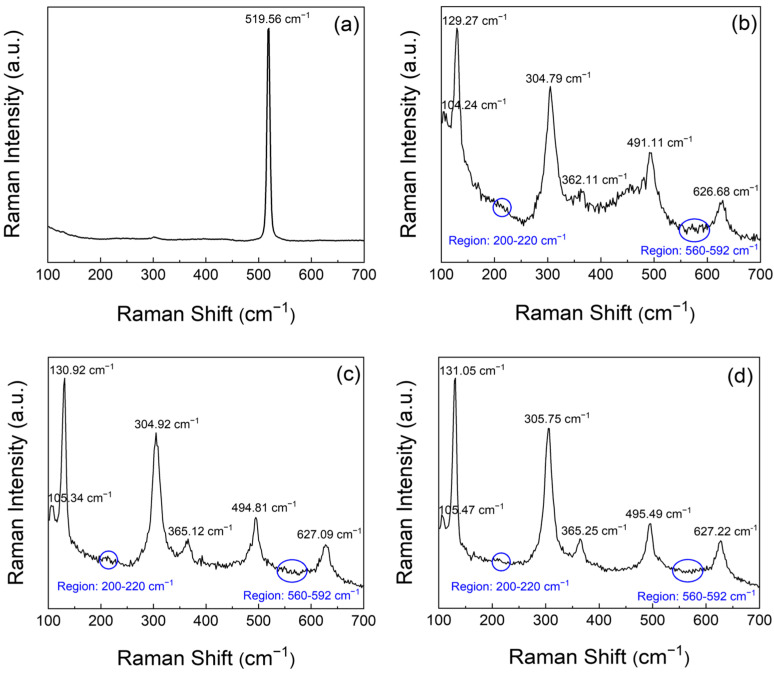
Raman spectra of the solution-processed films fabricated through (**a**) prebaking at 80 °C, thermal annealing at temperatures of (**b**) 350, (**c**) 450, and (**d**) 550 °C.

Figure 4 illustrates the results of Raman spectroscopic analysis of the solution-processed In_2_O_3_ thin films. Micro-Raman spectroscopy was performed using a Raman spectrometer equipped with a 532 nm excitation source under backscattering geometry. As shown in Figure 2c, the Raman spectra exhibit trends that align with the previously observed optical and crystallographic features, and reveal a clear distinction between the prebaked film at 80 °C and those subjected to thermal annealing at 350, 450, and 550 °C. The pre-baked film exhibits a single broad Raman peak centred at approximately 519.56 cm^−1^, as illustrated in Figure 4a, whereas the film annealed at 350 °C displays multiple distinct peaks at 104.24, 129.27, 304.79, 362.11, 491.11, and 626.68 cm^−1^, as shown in Figure 4b. In general, a shift in the Raman peaks towards higher wavenumbers is indicative of compressive stress. Thus, these spectral changes are indicative of the increased internal stress induced by thermal annealing [19]. This observation is consistent with the agglomeration of nanocrystalline grains in the prebaked film, as shown in Figure 2a,b. Such grain agglomeration becomes more pronounced following annealing at 350 °C. In_2_O_3_ typically crystallizes in a cubic bixbyite structure, belonging to the Ia-3 (T_h_^7^) space group, wherein the vibrational modes with A_g_, E_g_, and T_g_ symmetries are Raman active, and the T_u_ modes are infrared (IR) active [20]. The Raman spectrum of the film annealed at 450 °C (Figure 4c) exhibits distinct vibrational modes at approximately 105.34, 130.92, 304.92, 365.12, 494.81, and 627.09 cm^−1^, which correspond well to the characteristic phonon modes of cubic In_2_O_3_ [21]. Similarly, the spectrum of the film annealed at 550 °C (Figure 4d) exhibits slightly upshifted peaks at approximately 105.47, 131.05, 305.75, 365.25, 495.49, and 627.22 cm^−1^, suggesting enhanced compressive stress induced by high-temperature annealing [6].

**Figure 5:**
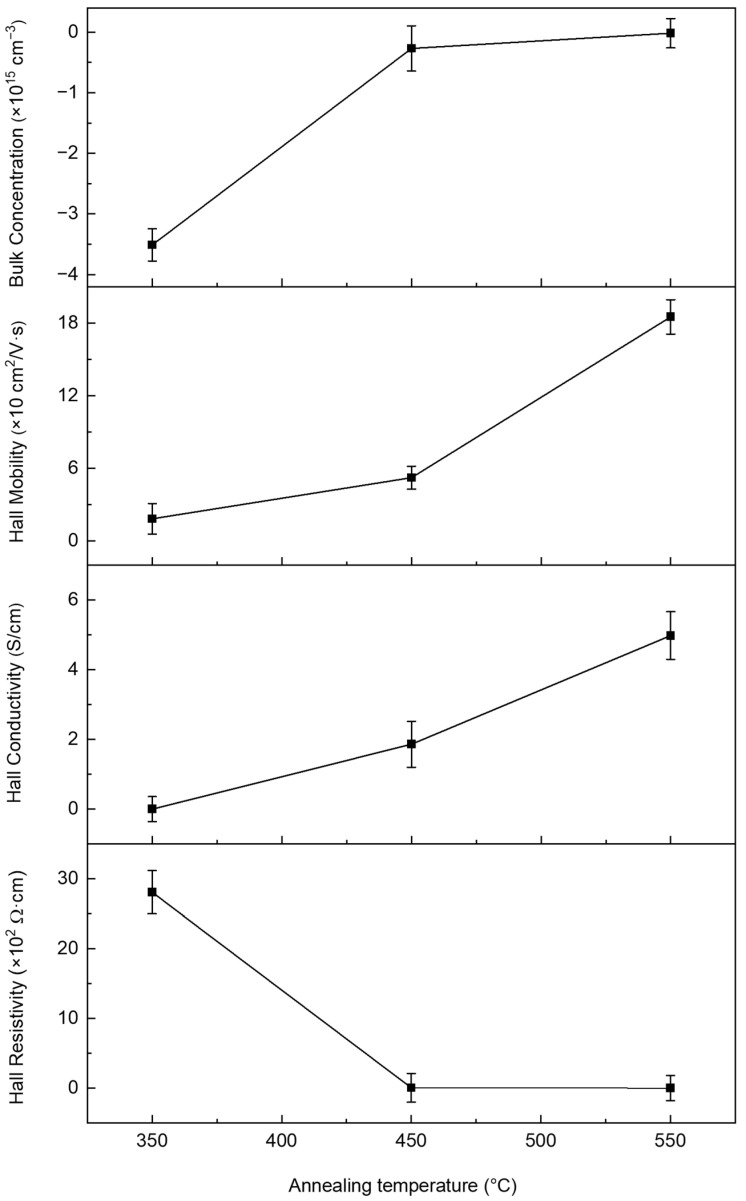
Hall effect measurement results showing the bulk concentration, hall mobility, conductivity, resistivity of the solution-processed films fabricated by thermal annealing at 350, 450, and 550 °C.

The peaks at approximately 131 and 305 cm^−1^ are attributed to the In–O bond vibrations and bending modes of the InO_6_ octahedra. Additionally, the satellite peaks observed at approximately 202, 568, and 588 cm^−1^ are indicative of residual stress in the thermally treated solution-processed films. Similar satellite features in the range of 572–582 cm^−1^ have also been reported in zinc oxide thin films and are typically attributed to intrinsic defects or oxygen vacancies induced by residual stress [22]. These oxygen vacancies are strongly associated with the blue emission characteristics of In_2_O_3_ [23]. The Raman peak observed at approximately 202 cm^−1^ remains unclear and warrants further investigation. Previous studies have suggested that this peak may arise from oxygen-vacancy-related local vibrational modes or strain/size-included activation of normally silent phonon modes [24]. The influence of the annealing temperature on the electrical properties of In_2_O_3_ thin films was investigated through Hall effect measurements. Herein, the results reveal systematic variations in key electrical parameters of the solution-processed In_2_O_3_ films with increasing temperature. As shown in Figure 5, a decreasing trend in resistivity was observed with increase in the annealing temperature. The key electrical parameters obtained from Hall effect measurements are listed in Table 1. At 350 °C, the resistivity was measured to be 2.81 × 10^3^ Ω·cm, indicating limited electrical conductivity. With increased annealing temperature, the resistivity decreased to 6.58 Ω·cm at 450 °C and further to 1.29 Ω·cm at 550 °C. These results suggest that high-temperature annealing reduces structural defects and enhances charge carrier mobility The Hall mobility increased significantly from 18.4 cm^2^/V·s at 350 °C to 52.3 cm^2^/V·s at 450 °C, and further to 185 cm^2^/V·s at 550 °C. This enhancement is attributed to the improved crystallinity at higher annealing temperatures, which facilitates more efficient charge transport [25].

The measured bulk concentration was −3.51 × 10^16^ cm^−3^ at 350 °C, where the negative sign indicates that electrons are the majority carriers, confirming the n-type nature of the In_2_O_3_ thin film. This value decreased to −2.69 × 10^15^ cm^−3^ at 450 °C and to −1.71 × 10^14^ cm^−3^ at 550 °C. This reduction in the bulk concentration is likely due to the annealing-induced passivation of oxygen vacancies, which act as electron donors in In_2_O_3_. Consequently, the electrical conductivity, which is inversely related to resistivity, exhibited a corresponding increasing trend with temperature varying from 1.18 × 10^−3^ S/cm at 350 °C to 1.86 S/cm at 450 °C, and further to 4.98 S/cm at 550 °C. This trend indicates that thermal annealing significantly enhances the electrical conductivity of In_2_O_3_ films by modifying their microstructural and defect characteristics. Among the examined conditions, the film annealed at 550 °C exhibited the highest electrical conductivity and Hall mobility, although a slight decrease in the bulk concentration was observed owing to defect passivation at elevated temperatures. These electrical improvements are primarily attributed to a reduction in the structural defects, particularly oxygen vacancies and indium interstitials, which are known to dominate the defect chemistry of In_2_O_3_. In_2_O_3_ is a well-established n-type semiconductor, in which oxygen vacancies and indium interstitials act as dominant donor-like defects. Therefore, the observed enhancement in the electrical conductivity and Hall mobility is attributed to the thermal activation of crystallization and the concurrent reduction in donor-type structural defects at elevated annealing temperatures [26].

**Figure 6:**
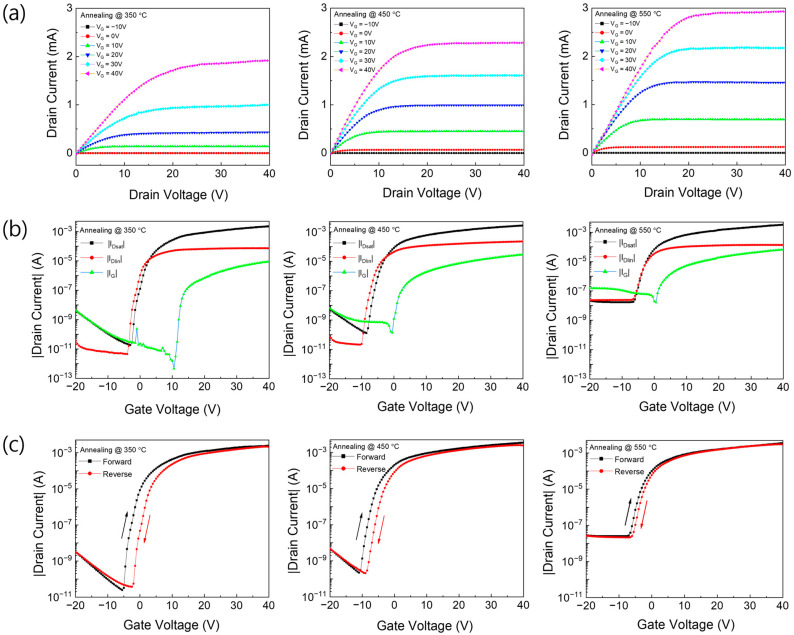
(**a**) Output (**b**) transfer and (**c**) hysteresis characteristics of the fabricated TFTs measured for thermal annealing at 350, 450, 550 °C.

Figure 6 illustrates the electrical characteristics of TFTs incorporating solution-processed In_2_O_3_ channel layers annealed at 350, 450, and 550 °C. The output characteristics (Figure 6a) were measured by sweeping the drain voltage from −20 V to 40 V in 0.5 V increments, with the gate voltage varied from −10 V to 40 V in 10 V steps. A progressive increase in the drain current was observed with increasing drain voltage. Moreover, higher annealing temperatures led to a notable enhancement in the overall drain current, indicating an improved channel conductivity. The device annealed at 350 °C exhibited relatively low drain currents, whereas the TFT annealed at 450 °C showed well-defined pinch-off behaviour and enhanced current saturation, indicating improved carrier modulation and interface quality. The device annealed at 550 °C exhibited the highest drain current, reflecting enhanced charge transport, likely due to increased crystallinity and reduced defect density in the In_2_O_3_ channel [27]. The transfer characteristics of the devices are shown in Figure 6b. The measurements were conducted in both the saturation and linear regimes. In the saturation regime, the gate voltage was swept from −20 V to 40 V, while the drain voltage was held constant at 40 V, whereas in the linear regime, the gate voltage was swept over the same range with the drain voltage maintained at 1 V. The primary electrical characteristics of the fabricated TFTs, including the field-effect mobility (µ), subthreshold swing (SS), and threshold voltage (Vth), were determined from the transfer curves measured in the saturation regime (V_DS_ = 40 V). The values of µ and SS were derived from the standard expressions for saturation current:

$$I_{DS}=\frac{1}{2}\mu C_{ox}\frac{W}{L}{\left(V_{GS}-V_{DS}\right)}^{2},\;\mu =\frac{2L}{WC_{ox}}{\left(\frac{d\sqrt{I_{DS}}}{d\;V_{GS}}\right)}^{2},\;SS={\left(\frac{d\log I_{DS}}{d\;V_{GS}}\right)}^{-1}$$

where C_ox_ represent the areal capacitance of the SiN_X_ dielectric layer, and W and L denote the channel width and length, respectively. 100 nm SiN_X_ dielectric has an areal capacitance of ~66 nF/cm^2^.

The mobility was extracted at the gate voltage corresponding to the maximum transconductance of the drain current, while SS was obtained from the steepest slop of the transfer curve in the subthreshold region. The threshold voltage was calculated by linearly extrapolating the square-root of I_DS_ in the saturation regime; the x-intercept of this tangent line, drawn at the point of maximum transconductance, defines Vth following the conventional approach for oxide TFTs [28]. The key electrical parameters obtained from the transfer characteristics are listed in Table 2. Device-to-device variations in the key electrical parameters in In_2_O_3_ TFTs are detailed in Supplementary Materials, Figure S1. Analysis of the transfer curves reveal that the TFT annealed at 450 °C exhibited the lowest threshold voltage −4.33 V, suggesting improved turn-on behaviour and enhanced energy efficiency for low-voltage applications.

The field-effect mobility (μ) of the TFT annealed at 450 °C is 4.28 cm^2^/V·s, which is comparable to that of the 350 °C device (4.32 cm^2^/V·s), suggesting minimal influence of annealing on intrinsic carrier transport. The sub-threshold swing slightly increases to 0.87 V/decade at 450 °C, but remains within an acceptable range for reliable switching behaviour. The on/off current ratio at 450 °C is 2.15 × 10^7^, which is slightly lower than that of the device annealed at 350 °C (1.08 × 10^8^), yet still sufficient for practical TFT applications. Hysteresis, an important parameter reflecting the interface quality and long-term reliability, exhibits a clear dependence on the annealing temperature. At 350 °C, the hysteresis voltage is 3.11 V, indicating a high density of charge trapping states at the channel/dielectric interface, which can adversely affect electrical stability during repeated operation. In contrast, the device annealed at 450 °C exhibits a reduced hysteresis voltage of 1.8 V, suggesting that interface defects and oxygen-related trap states are significantly suppressed at this temperature Passivated indium oxide [9]. The device annealed at 550 °C demonstrates the lowest hysteresis voltage (0.92 V).

However, this is accompanied by a markedly degraded on/off current ratio of 3.5 × 10^4^. This deterioration can be attributed to the elevated electrical conductivity and increased leakage current induced by high-temperature annealing. The relatively high gate leakage current observed in the experimental device can be attributed to the unfavourable band alignment of the p-type Si/SiN_X_/n-InO stacked gate electrode. Under a positive gate bias, the p-Si/n-InO interface behaves similarly to a forward-biassed junction, promoting carrier tunnelling through the SiN_X_ dielectric and thereby increasing the leakage current. In contrast, when an n-type Si gate is used, this forward-bias condition is eliminated, resulting in a significant reduction in the leakage current. This behaviour is qualitatively consistent with previous studies on oxide TFTs employing SiN_X_ gate dielectrics, which investigated the origins of unusual gate currents and the factors controlling their low [29]. As shown in Figure 5, the electrical conductivity of the In_2_O_3_ film increases with increasing annealing temperature, possibly resulting in an elevated off-state current, and consequently, a reduced on/off current ratio [1]. Considering the overall electrical performance including hysteresis, threshold voltage, field-effect mobility, and on/off ratio, the 450 °C annealing condition is determined to be the most optimal thermal treatment for solution-processed In_2_O_3_-based TFTs. This condition provides a well-balanced combination of low threshold voltage, stable field-effect mobility, suppressed hysteresis, and adequate on/off current ratio.

**Figure 7:**
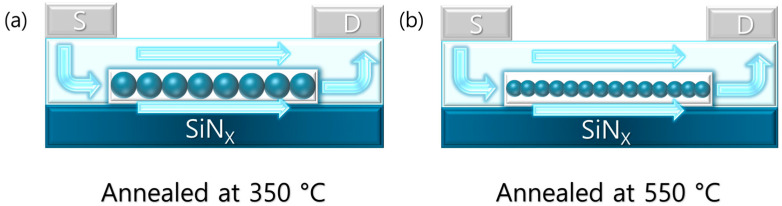
Channel formation at (**a**) 350 °C and (**b**) 550 °C with interfacial voids enlarging SS but reducing hysteresis.

Raman spectroscopy confirmed that increasing the annealing temperature led to agglomeration of In_2_O_3_ grain and overall improvement in crystallinity. However, this enhancement in crystallinity can simultaneously increase the distribution of void-like defects near the interface and grain boundaries, which may hinder charge transport during the initial channel formation and thereby deteriorate the SS. As the gate voltage increases, the channel extends upward by the electric field, and carriers predominantly flow through the upper crystalline region where fewer traps exist. SS analysis thus reflects the combined effect of both fast and slow traps, whereas hysteresis primarily captures traps that can respond within the sweep time. The calculated values of *N_it_* obtained from both methods are summarized in Table 3, based on the extraction approaches reported in previous studies [30,31]. Figure 7 illustrates the channel formation at 350 °C and 550 °C, indicating that interfacial voids contribute to an increased subthreshold swing (SS) while reducing hysteresis. Ultimately, even with improved crystallinity, the interfacial conditions and defect distribution still play a critical role in determining the electrical stability and reliability of the devices. Therefore, further studies focusing on the contact properties and energy distribution of defects at the semiconductor–insulator interface will be essential.

## 4. Conclusions

In this study, the influence of annealing temperature on the optical, crystallographic, and structural properties of solution-processed In_2_O_3_ thin films was systematically investigated. These findings underscore the critical importance of precisely controlling the relationship between annealing-induced structural variations and the resulting electrical characteristics to achieve optimized performance in In_2_O_3_-based TFT devices. To further clarify the correlation between the hysteresis behaviour and structural/electrical properties of solution-processed oxide semiconductors, additional systematic investigations are recommended. In particular, the effects of different annealing atmospheres such as ambient air, oxygen, and nitrogen-rich environments should be examined. As hysteresis is closely associated with trap states, interface defects, and oxygen vacancy concentrations, the quantitative analysis of defect evolution under controlled atmospheres can help establish optimal processing protocols for reliable and stable device operation. Beyond optimizing device performance at the 450 °C post-annealing condition, this study emphasizes stability aspects such as hysteresis behaviour. By extending the discussion to reliability, our findings provide valuable insights not only into thin-film microstructure and electrical performance but also into the long-term device stability that is essential for practical applications and future commercialization.

## Figures and Tables

**Table 1:** The electrical properties of In_2_O_3_ thin films with different annealing temperatures.

Annealing Temperature	Hall Resistivity (Ω·cm)	Bulk Concentration (cm^−3^)	Hall Mobility (cm^2^/V·s)	Hall Conductivity (S/cm)
350 °C	2.81 × 10^3^	−3.51 × 10^16^	18.4	1.18 × 10^−3^
450 °C	6.58	−2.69 × 10^15^	52.3	1.86
550 °C	1.29	−1.71 × 10^14^	185	4.98

**Table 2:** Characterization of the electrical properties of solution-processed In_2_O_3_ TFTs.

Annealing Temperature	Threshold Voltage (V)	Field-Effect Mobility (cm^2^/V·s)	Sub-Threshold Swing (V/dec)	On/Off Ratio	Hysteresis (V)
350 °C	0.97	4.32	0.42	1.08 × 10^8^	3.11
450 °C	−4.33	4.28	0.87	2.15 × 10^7^	1.8
550 °C	−4.21	4.97	1.86	3.5 × 10^4^	0.92

**Table 3:** Interfacial trap densities of In_2_O_3_ TFTs extracted from SS (*D_it_*) and ΔV_T_ (*N_it_*) at different annealing temperatures.

Annealing Temperature	Subthreshold Swing (V/dec)	Interfacial Trap Density Subthreshold Swing (cm^−2^ eV^−1^)	Threshold Voltage Shift (V)	Interfacial Trap Density Threshold Voltage Shift (cm^−2^)
350 °C	0.42	1.4 × 10^11^	3.11	1.28 × 10^12^
450 °C	0.87	3.1 × 10^11^	1.8	7.42 × 10^11^
550 °C	1.86	7.0 × 10^11^	0.92	3.79 × 10^11^

## Data Availability

The research data presented in this study are available upon request from the corresponding authors.

## Supplementary Materials

The following supporting information can be downloaded at: https://www.mdpi.com/article/10.3390/mi16101091/s1, Figure S1: Device-to-device variation in the key electrical parameters in In_2_O_3_ TFTs for 12 devices.
