# Supplementary material for: Optimizing Annealing Temperature for Enhanced Electrical Performance and Stability of Solution-Processed In2O3 Thin-Film Transistors
Source: Micromachines (Basel). 2025 Sep 26;16(10):1091. doi: 10.3390/mi16101091 (PMC12566397; doi:10.3390/mi16101091)
Supplement: Supplementary file 1 [file micromachines-16-01091-s001.zip › micromachines-3857565-supplementary.pdf]

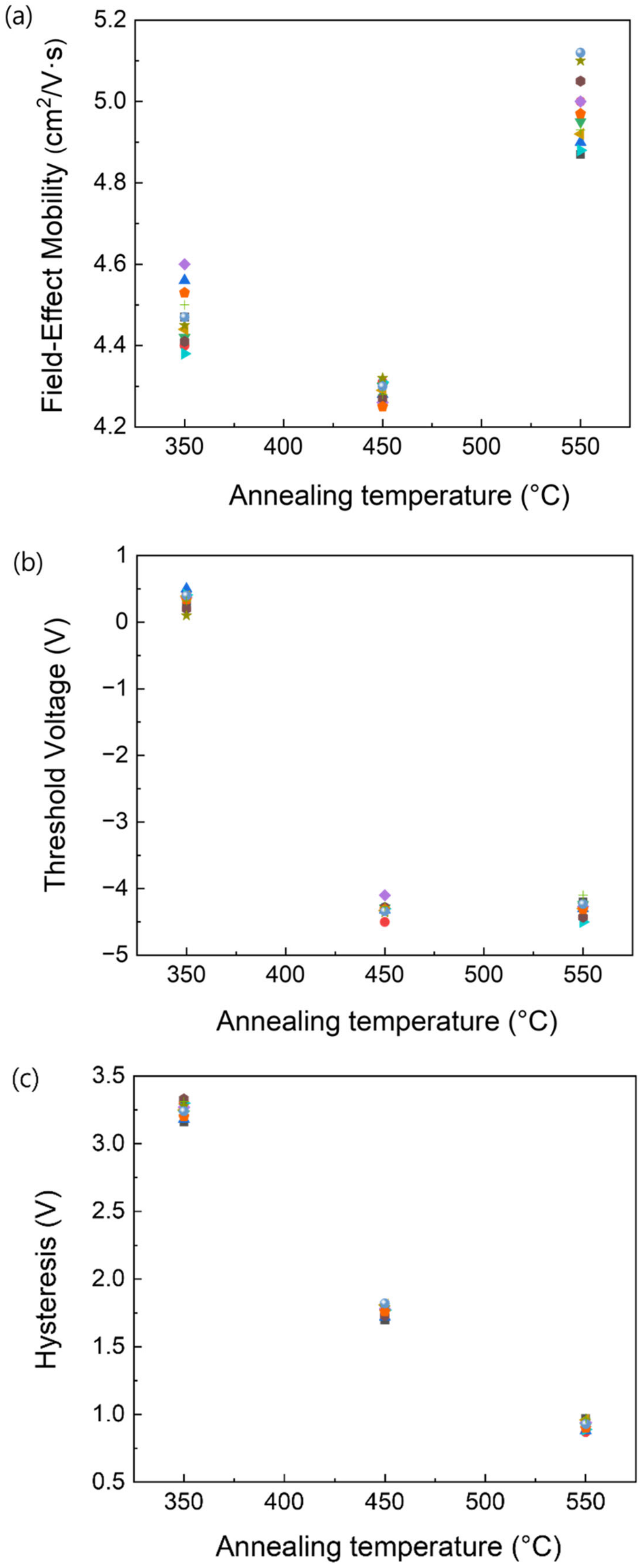

Figure S1. Device-to-device variation of the key electrical parameters in In_2O_3 TFTs: (a) field-effect mobility, (b) threshold voltage, and (c) hysteresis. The plots present the distributions obtained from 12 individual devices, highlighting the reproducibility of the fabricated transistors.
